# Surgical management of life threatening events caused by intermittent aortic insufficiency in a native valve: case report

**DOI:** 10.1186/1749-8090-5-94

**Published:** 2010-10-29

**Authors:** Mary H Martin, Stanton B Perry, James V Prochazka, Frank L Hanley, Norman H Silverman

**Affiliations:** 1 Department of Pediatric Cardiology, Lucile Packard Children's Hospital, Stanford University Medical Center, 750 Welch Road - Suite 305, Palo Alto, California 94304, USA; 2 Department of Cardiology and Cardiothoracic Surgery, Children's Hospital Central California, 9300 Valley Children's Place, Madera, CA 93636, USA; 3 Department of Pediatric Cardiothoracic Surgery, Lucile Packard Children's Hospital, Stanford University Medical Center, 750 Welch Road - Suite 305, Palo Alto, California 94304, USA

## Abstract

We describe a case of a patient admitted with apparent life threatening events characterized by hypotension and bradycardia. The patient was ultimately found to have intermittent severe aortic insufficiency. Upon surgical exploration, abnormalities were discovered in the aortic valve, which had a small left coronary cusp with absence of the nodulus of Arantius. Following surgical repair of the valve, aimed at preventing the small cusp from becoming stuck in the open position, the patient has remained episode free for over one year.

## Background

Apparent life threatening events (ALTEs,) characterized by a combination of apnea, decreased muscle tone, and color change, remain a challenge for general and subspecialty pediatricians. The possibility of sudden infant death syndrome (SIDS,) though not necessarily related to ALTEs, leads to an extensive work-up for these events. In the approximately 50% of ALTE work-ups that do reveal a diagnosis, 98% of these have gastrointestinal, neurologic, or respiratory etiologies[1]. However, in part because of the large number of idiopathic ALTEs, cardiologists are frequently consulted in these cases when the reflux and neurologic work-ups are unrevealing. The commonly accepted cardiac causes are limited to arrhythmias, cardiomyopathies, and structural disease, most of which can be diagnosed by echocardiogram or EKG monitoring. We present a case of life-threatening events caused by intermittent aortic insufficiency, and a surgical solution to these events.

## Case Presentation

CV was a term infant who presented to another hospital at 5 weeks of age following an episode of turning grey and unresponsive. Elevated troponin was noted during extensive work-up of the event. The only abnormal finding was on coronary angiography, showing an irregularity in the LAD suspicious for an intramural coronary. Lacking any other explanation for the events, he underwent surgical unroofing of a myocardial bridge at 6 weeks of age. However, several weeks later he experienced another similar episode requiring CPR. Echocardiography after the event showed normal systolic function, no valvar insufficiency, but raised the question of diastolic dysfunction. Catheterization at this time showed LVEDP of 22 mmHg. He was transferred to our institution on 9/19/08 for transplant evaluation as it was thought that his episodes might have been related to poor diastolic function.

**Figure 1:**
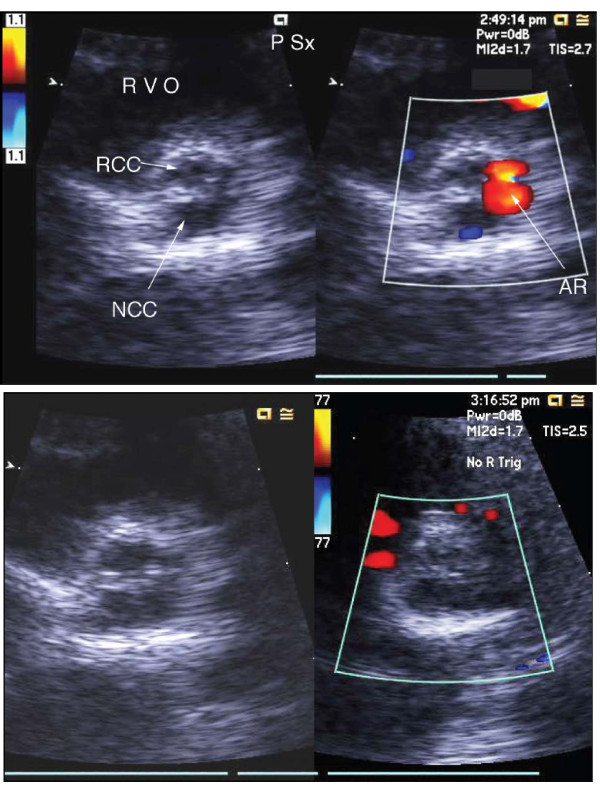
**Severe aortic insufficiency seen on transthoracic echocardiography during catheterization**. Severe aortic insufficiency is noted through the area of the left coronary cusp. Aortic insufficiency is terminated by placement of catheter into left coronary cusp.

On arrival to our institution, he had another acute event again characterized by hypotension, followed by desaturation and bradycardia. Echocardiogram at our institution was normal. A planned pre-transplant catheterization procedure was expedited to re-investigate the coronary arteries to look for a possible cause for his acute decompensation. At cardiac catheterization, prior to any attempt to cross the aortic valve, he had another event. Aortic angiography during the event showed significant aortic regurgitation. Echo confirmed severe aortic regurgitation, which appeared to be predominantly through the area of the left coronary cusp. The regurgitation stopped after insertion of a catheter retrograde into the left coronary cusp, and the systolic blood pressure increased immediately from 45 to 100 mmHg, (Figure 1). The LVEDP had declined to 6 mmHg, and the coronaries were normal. We undertook surgical exploration for possible aortic valve repair.

**Figure 2:**
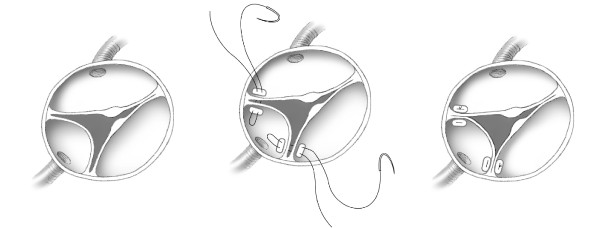
**Artist's depiction of intraoperative appearance and surgical repair of aortic valve**. The left coronary cusp is small and the nodulus of Arantius is absent on that cusp. The repair included placement of a prolene pledgeted suture near each of the commissural posts of both the left-right and the left-non commissures, creating partial fusion of these commissures.

An intraoperative transesophageal echocardiogram (TEE) showed no evidence of aortic insufficiency. On direct inspection the aortic valve was trileaflet, but the left coronary cusp was abnormally small and made up only approximately 20% of the circumference of the aortic annulus. While the right and non-coronary cusps had normal appearing nodules of Arantius, the nodule was absent in the left coronary cusp. The intraoperative hypothesis was that the absence of the nodule of Arantius on the left cusp allowed that cusp to become intermittently stuck in the open systolic position, creating acute severe AI and possibly also left main coronary artery insufficiency. A single 6-0 prolene pledgeted suture was placed near each of the commissural posts of both the left-right and the left-non commissures, creating partial fusion of these commissures, (Figure 2). The goal of this maneuver was to partially restrict the motion of the left cusp, preventing it from opening completely and adhering to the wall of the left sinus of Valsalva. Following the operation, there was minimal stenosis at the level of the aortic valve with a peak gradient of 21 mmHg by echo. The patient made an uneventful recovery, and was discharged on postoperative day #8. At one-year follow-up, the patient has not had any further life threatening events, and echo showed normal biventricular function with a 28 mmHg peak gradient across the aortic valve.

## Conclusions/Discussion

We could find no reports of intermittent aortic valve regurgitation in a native aortic valve. However, there are has been significant work, both in vitro and in vivo to look at the function of the aortic valve in normal and abnormal physiologic states. Much of this work was done by B. J. Bellhouse, who showed in a pulsatile water-filled rigid-walled tunnel that approximately 75% of aortic valve closure occurs during forward flow, and is contingent upon vortices which form during systole in the sinuses of Valsalva. The flow pattern is dependent upon the center (tip) of the cusp projecting about 2 mm into the sinus, with the corners (sides) of the cusp projecting slightly into the aorta, such that flow enters the sinus during systole at the center of the cusp, and exits the sinus on both sides of the cusp [2,3]. The vortex creates a pressure that is equivalent to the radial pressure in the aorta during peak forward flow, such that a stagnation point is reached during which the cusps are immobile and the flow in the aorta is linear. During the deceleration phase of systole, the pressure created by the vortex transiently exceeds that of the linear flow in the aorta, and the valve begins to close. If flow does not enter the sinus at the cusp tip, then the vortex, necessary to counter the pressure in the aorta to prevent collapse of the cusp into the sinus of Valsalva, cannot be established [4]. This phenomenon is most likely to occur in the left sinus because the left cusp is generally the smallest of the 3 cusps of the aortic valve in the general population, both in surface area and in weight [5,6].

We postulate that the abnormal left coronary cusp and the absence of the nodulus of Arantius intermittently prevented formation of this vortex during systole in our patient. Without the existence of the vortex, the valve leaflet was forced against the sinus, leading to severe aortic regurgitation until the valve leaflet again became mobile. The stimulus that dislodged the leaflet from the sinus was, at least in one case, a catheter, but it could in theory also be severe hemodynamic alterations such as those associated with CPR. We postulate that this process lead to significant left coronary artery ischemia, both secondary to AI and to physical obstruction of the left coronary os. This phenomenon accounts for the elevated troponin that had occasionally followed his life-threatening events, and also explains his sudden decrease in blood pressure which seemed to initiate at least one of these events, documented by both hemodynamic measure at catheterization, and echocardiography.

There are many patients who present for surgical repair due to abnormal valves with small leaflets that have absent or effaced noduli of Arantius, and yet the presentation of intermittent severe aortic insufficiency has not before been reported. It is unclear why this patient presented in this way, while others manifest their valvar abnormalities with chronic aortic stenosis or insufficiency. We can only hypothesize that this particular valve leaflet was the exact shape and size that would lead to intermittent fixation of the leaflet in the open position, causing this unusual presentation.

We consider that this near sudden death event may be an unheralded mechanism and that cardiologists, surgeons, and pathologists should note the relationship of the sinus of Valsalva to the coronary cusp in other episodes of unexplained severe life threatening events. The absence of events in this child in the year since his operation supports our theory for the etiology of these events, and also suggests that his operation was successful in preventing further events.

## Competing interests

The authors declare that they have no competing interests.

## Authors' contributions

MM reviewed the case, conducted a review of the literature, and wrote the case report. SP diagnosed the patient with intermittent aortic insufficiency in the catheterization laboratory. JP provided patient follow-up and data. FH performed the operation described and participated in the literature review. NS confirmed the patient's diagnosis with echocardiography, conducted a review of the literature, supervised the writing of the manuscript, and revised the manuscript, contributing important intellectual content. All authors read and approved the final manuscript.

## Consent

Written informed consent was obtained from the patient's parents for publication of this case report and any accompanying images. A copy of the written consent is available for review by the Editor-in-Chief of this journal.

## References

[B1] Farrell PA Weiner GM Lemons JA SIDS, ALTE, apnea, and the use of home monitors Pediatrics in Review 2002 23 1 3 9 10.1542/pir.23-1-3 11773587

[B2] Bellhouse BJ Talbot L The Fluid Mechanics of the Aortic Valve J Fluid Mech 1969 35 721 735 10.1017/S0022112069001406

[B3] Yoganathan AP Lemmon JD Ellis JT Bronzino JD Heart Valve Dynamics The Biomedical Engineering Handbook 2000 Chapter 29 2 CRC Press in cooperation with IEEE Press

[B4] Handke M Heinrichs G Beyersdorf F Olschewski M Bode C Geibel A In vivo analysis of aortic valve dynamics by transesophageal 3-dimensional echocardiography with high temporal resolution Journal of Thoracic and Cardiovascular Surgery 2003 1412 1419 10.1016/S0022-5223(02)73604-0 12830062

[B5] Ciotti GR Vlahos AP Silverman NH Morphology and function of the bicuspid aortic valve with and without coarctation of the aorta in the young American Journal of Cardiology 2006 98 8 1069 1102 10.1016/j.amjcard.2006.05.035 17027579

[B6] Stewart WJ King ME Gillam LD Guyer DE Qeyman AE Prevalence of aortic valve prolapse with bicuspid aortic valve and its relation to aortic regurgitation: a cross-sectional echocardiographic study American Journal of Cardiology 1984 1277 1282 10.1016/S0002-9149(84)80080-6 6507297

